# Injuries to the Spinal Cord

**Published:** 1912-08

**Authors:** Frank K. Boland

**Affiliations:** Atlanta, Ga.; Professor of Operative and Clinical Surgery, Atlanta School of Medicine


### INJURIES TO THE SPINAL CORD.

By Frank K. Boland, M. D., Atlanta, Ga.

*Professor of Operative and Clinical Surgery, Atlanta School of Medicine.*

This paper consists in the recital of seven cases of spinal cord injury in my practice, and the lessons which I have learned from them. Fortunately, the condition is rare. Two of my cases were seen in private practice, and five in my hospital work. Laminectomy was done in five cases.

The first case was due to a gun-shot wound. The bullet pierced the apex of the right lung and lodged in the spinal canal opposite the third dorsal vertebra. No radiograph was made and the diagnosis was reached from the history of the accident and from the physical signs. The patient developed pneumonia on the second day, and laminectomy was performed under cocaine on the third day. The bullet, which had severed the cord completely, was removed, and the wound closed with drainage. No attempt was made to bring the ends of the cord together, because the physiologists told us at that time, ten years ago, and still tell us, that it is impossible to cause regeneration of divided nerve tissue. In spite of this, DaCosta cites three cases where suture of the spinal cord was done with remarkable success. Of course, this does not mean that perfect restoration of function was obtained, but the patients did regain very good control of the bladder and rectum, and were able to stand and take a few steps, and had life prolonged indefinitely. My case, which came to me during hospital interneship, was the only one I have ever had in which there was opportunity to suture the cord, and I will never cease to regret my failure to do so. The patient, a thirteen-year-old boy, lived for three weeks after the operation, and died of sepsis from bed-sores.

The second case was a fracture-dislocation of the fourth and fifth cervical vertebrae, the patient having been thrown from a buggy against a telegraph pole. Deformity and paralysis were so well-marked that operation was done immediately without waiting for an X-Ray. Only very gentle manipulations were attempted in an effort to reduce the dislocation before making an incision. Some text-books give careful instructions as to the manipulations to be used in reducing dislocated vertebrae, but I believe any method is useless and dangerous. This case was a typical one where "the operation was a success, but the patient died." The displacement of the fourth vertebrae forward on the fifth, with fracture of both, was found and relieved. So far as could be told with the naked eye, the cord was not lacerated or bruised. The wound was closed and the patient encased in a plaster jacket holding his neck straight and in extreme extension. He rallied quickly from the anaesthetic, and within twelve hours had better control of his bladder and rectum. Sensation was returning in his lower extremities, and he developed an appetite and said he felt well. But on the third day, just as I entered his room and started toward his bed, his face suddenly turned inky black and he died instantly. An autopsy could not be obtained, but he must have died of a hemorrhage into the pons or medulla.

I next had a case of crush of the third, fourth and fifth dorsal vertebrae, well shown by the X-Ray. Laminectomy was done six hours later, and pressure removed from a badly bruised cord. The patient died before he could be gotten from the table. The fourth case was similar to this, except that the injury was lower down, and the patient survived the operation several hours, though he never regained consciousness. I believe that shock and hemorrhage contributed to the early death of these cases. Considerable loss of blood is unavoidable in such a large incision, and the shock of cutting through the laminae of three or four vertebrae must be considerable. Very strong bone-biting forceps, such as those of Dr. Hudson, should be used for this purpose, and the work completed as rapidly as consistent with thoroughness. In one case I tried the English method of turning back a flap of skin and muscle before exposing the bone, but I think the loss of blood this way is greater than with the straight median incision.

Case five taught the value of waiting when signs of a definite lesion are absent, notwithstanding that the violence of the patient's accident could have accounted for any degree of injury to the tissues. He was a telephone lineman, who had fallen twenty-five feet to the pavement. No bones were broken, but there was total paralysis from the dorso-lumbar junction downward. Since the radiographs were negative and no displacement could be detected, the so-called "expectant" policy was adopted. Within a few days the paralysis began to disappear, and within three weeks the patient left the hospital practically well. There is much debate as to the existence of the condition we call concussion, but it is my opinion that this was a case of concussion, or shock, of the spinal cord, otherwise the patient would not have mended so promptly.

The sixth case was unsatisfactory in that death occurred without the discovery of an appreciable lesion. The case, which was the result of a fall from a second-story window, was ten days old when operated upon, and had total paralysis from the cervico-dorsal junction downward. A radiograph could not be obtained. The laminae of the sixth and seventh cervical and first three dorsal vertebrae were removed, and the cord exposed without seeing much injury to the vertebrae or to the cord. It must be remembered that the injury is usually higher than the line of paralysis, and perhaps we should have removed even more laminae, or perhaps the injury was present but not apparent to the naked eye. Bailey, in his much-quoted book, "Diseases of the Nervous System Resulting from Accident and Injury," states that in many cases the cord is compressed, paralysis produced, and the compression finally removed without leaving any macroscopical trace. This is given as an argument against operation, but I do not see how we can tell the condition of the cord until we inspect it. Again, the compression might be due to blood-clot, which can be relieved by operation. Even if damage to the cord does remain after the removal of pressure, certainly the cord has some chance to regenerate with the pressure gone, and it has none with the pressure still present.

The last case of injury to the spinal cord followed a stab-wound with a three-inch pocket-knife blade between the seventh and eighth dorsal vertebrae. The patient, a robust boy of fifteen, fell at once, completely paralyzed from the waist downward. The knife-blade was extracted with difficulty. The wound was opened at once down to the vertebrae, cleaned and drained. No displacement of bone could be felt, and radiographic views from two sides showed no bony displacement or foreign body. With this evidence, and with the conviction that the knife-blade could not have severed the entire cord, and with the remembrance of my 100% mortality following operations, I decided to wait on this case. One side-point: the interne reported that there was incontinuance of urine and no need to catheterize. Examination showed that there was incontinuance, but it was an overflow, and the bladder was distended with twenty ounces of urine. Catheterization had to be resorted to for about three weeks, and cystitis was hastened and augmented by the presence of an acute gonorrhea from which the patient was suffering at the time he was hurt. For about a month the history of this case made me feel very well over my decision to be conservative. The patient regained almost complete control of his rectum, incontinence having existed for the first two or three weeks. Though his urine dribbled at times, he could empty his bladder at will, and the catheter was discontinued. The area of anaesthesia moved from a point just below the umbilicus down to Poupart's ligament, a distance of six inches. Here it stopped, and for the last two weeks he failed rapidly in every way, and finally died seven weeks after receiving the injury. According to Bailey this case followed the usual course without operation, in that there was very hopeful improvement for about a month and then rapid decline. He attributes the improvement to the absorption of small blood-clots in and around the cord. I think the immediate cause of the death of this patient was exhaustion following a several weeks' seige of sepsis from bed-sores. Of course, in this series of cases bed-sores received every possible attention in prevention and treatment.

In suspected injury to the spinal cord a radiograph should be made at once to determine the presence of fracture or dislocation or foreign body. Where deformity is very evident this may not be necessary for diagnosis, but it is always valuable as a record. When pressure symptoms exist and compression of the cord is probable, operation should be done as soon as constitutional shock disappears. Of course, concussion or shock of the cord must first be eliminated. Symptoms due to this cause will soon begin to disappear. Fracture of a process may be present without injury to the cord, but hardly fracture of a lamina. It is for injury to the cord that we operate and not injury to the bone. There might be cases, however, of fracture without cord symptoms where operation was indicated to prevent later cord injury. Incision must be free. Plenty of laminae must be cut, the dura opened, and the cord thoroughly inspected. Freshen the ends of a severed cord and suture them together. If the tension be too great on account of the amount of cord which has been destroyed, one writer has suggested that a vertebrae or two might be removed. Tension can be relieved by a large cast holding the trunk in extension. Unless there is some contraindication from injury to other viscera or from disease, gun-shot wounds and stab-wounds of the spinal cord, when properly diagnosed, and simple concussion eliminated, should be opened up at once, cleaned, and the damage to the cord repaired, if possible. It is better to operate on a simple concussion than to overlook a cord lesion which might have been relieved.

Injury to the spinal cord which does not at once crush out life is about the worst physical misfortune that can befall mankind, and the mortality will always be enormously high, whatever plan of treatment is pursued. If we study large series of cases, however, and not depend for guidance upon a few isolated ones, I think we will reach the conclusion that operative interference should be the usual, rather than the occasional course of treatment.

